# Lived experience of Iranian pre-hospital medical staff during the COVID-19 pandemic: a descriptive phenomenological study

**DOI:** 10.3389/fpsyg.2023.1230892

**Published:** 2023-12-21

**Authors:** Mehdi Jafari-Oori, Manigeh Dehi, Abbas Ebadi, Seyed Tayeb Moradian, Hajar Sadeghi, Mojtaba Jafari

**Affiliations:** ^1^Atherosclerosis Research Center, Faculty of Nursing, Baqiyatallah University of Medical Sciences, Tehran, Iran; ^2^Maragheh University of Medical Sciences, Maragheh, Iran; ^3^Behavioral Sciences Research Center, Life Style Institute, Faculty of Nursing, Baqiyatallah University of Medical Sciences, Tehran, Iran; ^4^University of Social Welfare and Rehabilitation Sciences, Tehran, Iran; ^5^Bam University of Medical Sciences, Bam, Iran

**Keywords:** COVID-19, lived experience, qualitative study, pre-hospital services, emergency medical staff

## Abstract

**Background:**

Pre-hospital medical staff faced numerous challenges during the COVID-19 pandemic. However, these challenges specific to pre-hospital services have not been thoroughly explored in Iran. This qualitative study aimed to examine the essence of pre-hospital care during the COVID-19 pandemic.

**Methods:**

This phenomenological study was conducted from June to August 2021 in Tehran, Iran. Semi-structured interviews were conducted with pre-hospital medical staff. Data analysis was performed using Colaizzi’s approach, and rigor was ensured by adhering to the consolidated criteria for qualitative reporting research.

**Results:**

A total of 17 pre-hospital medical staff were interviewed, and five themes were extracted from the data: workload and resilience, damage, lack of control, under preparedness, and post-traumatic growth. These themes highlight the resilience demonstrated by pre-hospital medical staff, who faced an unprecedented crisis with limited preparedness and significant damage.

**Conclusion:**

The findings of this study indicate that pre-hospital medical staff in Iran encountered challenges during the COVID-19 pandemic due to a lack of preparedness and substantial damage. Despite these adversities, the participants exhibited resilience and experienced post-traumatic growth. The study emphasizes the importance of proper planning and preparedness to enhance the resilience of emergency medical services during pandemics. Furthermore, the results underscore the need to address the challenges faced by pre-hospital medical staff and improve the quality of care provided to patients during crises such as the COVID-19 pandemic.

## 1 Introduction

There were over 570 million global COVID-19 cases and 6 million fatalities as of July 24, 2022 (WHO, 2020a). During the same period in Iran, there were 7,319,322 confirmed cases and 141,650 reported deaths (WHO, 2020a). The Delta variant of COVID-19 has resulted in an unprecedented surge in the death rate across all age groups, with an average of 750 deaths per day, posing significant challenges to the prevailing conditions (Shakibnia et al., 2021).

The COVID-19 pandemic has posed unprecedented challenges for prehospital medical staff globally (WHO, 2020b; Shakibnia et al., 2021; Heidari et al., 2023), including those in Iran (Heidari et al., 2023). Reluctance to care for highly infectious patients has led to adverse events during prehospital transport, even for short distances, potentially resulting in life-threatening situations (Baru et al., 2022). Studies have revealed a 80% increase in out-of-hospital cardiac arrests during the peak of the pandemic in certain regions (Fothergill et al., 2021). Additionally, the disruption of essential health services during epidemics can contribute to higher mortality rates, surpassing the direct deaths attributed to the disease itself (Neyazi et al., 2023).

Prehospital medical staff’s experiences during the COVID-19 pandemic are shaped by occupational hazards and psychological challenges They navigate complex environments, swiftly adapting to escalating demands (Mohammadi et al., 2021). Challenges include infection risk, personal protective equipment (PPE) shortages, exposure to distressing scenes, patient mortality, and the psychological toll of witnessing suffering and fearing transmission to loved ones (Jafari et al., 2019; Fatahi et al., 2022; Mohammadi et al., 2022).

Prehospital medical staff worldwide have faced significant job stress during the COVID-19 pandemic (Dami and Berthoz, 2020; Shahzad et al., 2020; Piotrowski et al., 2021; Nyashanu et al., 2022; Tune et al., 2022). COVID-19 has caused shifts in demand for emergency medical services in Canada (Ferron et al., 2021), an increase in nationwide EMS responses and deaths attended by EMS in the United States (Lerner et al., 2020), severe overload in Denmark’s emergency dispatch facility (Jensen et al., 2020), and overwhelming prehospital services in Iran with a surge in confirmed cases and record-breaking daily dispatches (Mohamadian et al., 2021). In Iran, prehospital services are overwhelmed with record-breaking 4,557 daily dispatches and a 347% surge in call volumes (Saberian et al., 2020).

While several international studies have examined the experiences of prehospital medical staff during the COVID-19 pandemic (Piotrowski et al., 2021; Nyashanu et al., 2022; Tune et al., 2022), limited research exists on the specific challenges faced by Iranian prehospital medical staff during the COVID-19 pandemic, particularly during the Delta wave (Hadian et al., 2022; Heidari et al., 2023). Hadian et al. highlighted the adverse impact of inadequate equipment and job overload on mental health and emergency care quality (Hadian et al., 2022), while Heidari et al. identified individual and systemic challenges requiring organizational planning and policy attention (Heidari et al., 2023). However, there is still a noticeable gap and limitations in understanding the lived experiences of Iranian prehospital medical staff within the unique context of their services, especially during the Delta wave.

Gaining an understanding of the lived experiences of Iranian prehospital medical staff could offers more valuable insights into their distinct challenges, support systems, and opportunities for enhancement. The prehospital services in Iran, primarily operated by governmental organizations like EMS and Red Crescent Society, as well as the private sector, confront unique obstacles such as resource limitations and geographical disparities. The prehospital services have been provided by male staff. Furthermore, our study may offer unique insights into the experiences of pre-hospital medical staff during the prevailing Delta wave in Iran, which has been relatively understudied compared to other health workers such as physicians (Liu et al., 2020), nurses’ (Ruiu, 2020), and family caregivers’ (Jafari-Oori et al., 2022). During the summer of 2021, the Delta wave of the COVID-19 pandemic in Iran was distinguished by the rapid transmission of the highly contagious Delta variant. This period witnessed a significant increase in cases, placing immense strain on healthcare systems and leading to a rise in hospitalizations and fatalities. The Delta variant’s heightened transmissibility and potential for more severe disease outcomes compared to earlier variants heightened alarm, prompting the implementation of targeted measures to curb its spread (Zali et al., 2022).

To bridge the above mentioned gap, it is essential to gain a more comprehensive understanding of the lived experiences of pre-hospital medical staff. Using qualitative methods like phenomenology provides valuable insights into the perspectives, emotions, and coping mechanisms of prehospital staff. This approach enables a deeper understanding of the unique challenges they face, informing targeted policy improvements and support systems that address their specific needs (Husserl, 2019). Therefore, the research question guiding this study is: “What are the lived experiences of Iranian pre-hospital medical staff during the Delta wave of COVID-19 pandemic?” By employing a descriptive phenomenological method, our aim is to delve into these experiences and make a valuable contribution to the existing literature.

## 2 Methods

### 2.1 Design and setting

The study utilized phenomenology as the qualitative approach to delve into the lived experiences of prehospital medical staff during the COVID-19 pandemic. Phenomenology was chosen because its purpose aligns with the aim of describing the essence of these experiences, capturing the subjective and nuanced aspects of the participants’ perceptions and interpretations (Bengtsson, 2016). Colaizzi’s phenomenological approach, in particular, was employed due to its emphasis on identifying shared experiences among participants. This method involves a rigorous analysis of interview data to uncover common themes and essential elements that represent the essence of the phenomenon under investigation (Colaizzi, 1977).

### 2.2 Participants

The study population comprised pre-hospital medical staff who provided pre-hospital services during the prevalence of the Delta variant of the virus. Purposive sampling was employed to select the participants, including both nurses and EMTs, in order to ensure maximum variation in their profession, education level, and years of experience. This variation encompassed different education levels (bachelor’s degree, associate’s degree, and master’s degree) and a range of experience from 4 to 25 years.

To implement purposive sampling and identify suitable participants, the researchers took an active approach. They collaborated with relevant institutions and utilized personal contacts to reach out to potential participants who met the specific criteria for maximum variation. The researchers clearly explained the purpose of the study and sought individuals who were willing to share their experiences as prehospital medical staff during the pandemic. By employing this strategy, the researchers aimed to capture a diverse range of perspectives and insights from participants with varying backgrounds and experiences in the field of prehospital care. Inclusion criteria for participants in this study were:

1. Pre-hospital medical staff working in Iran during the COVID-19 pandemic.
2. Nurses and pre-hospital medical staff involved in pre-hospital services.
3. Individuals with different levels of education (e.g., bachelor’s degree, associate’s degree, or master’s degree) in nursing or prehospital emergency care.
4. Participants with a minimum number of years of experience in pre-hospital care (e.g., 1 year or more).
5. Willingness to share their experiences and perspectives regarding the challenges and lived experiences during the COVID-19 pandemic.

Pre-hospital medical staff who did not complete the interview or provide sufficient data for analysis were excluded.

### 2.3 Data collection

The interviews were conducted between June and August of 2021. The data collection process involved in-depth interviews conducted by two authors, M.J.O (an assistant professor) and H.S. (a PhD student). The interviews began with open-ended questions and gradually progressed to more specific inquiries, as outlined in Table 1. Prior to each interview, the research team emphasized the significance of the study and provided an overview of the research conditions. The interviews were recorded, and their durations varied, with an average length of approximately 50 min. The interviews ranged from 40 to 60 min in duration. All interviews took place in EMS centers in a quiet room at the end of the staff’s shift. Throughout the interviews, field notes were taken to ensure accurate and comprehensive documentation of the responses. There were no interruptions during the interviews, and the data collection proceeded without any disruptions. In order to protect the participants’ privacy, we assigned them code names such as p1, p2, and so on.

**Table 1:** The semi-structured interview form.

Main questions	1. May you describe your pre-hospital emergency care during the COVID-19 pandemic? 2. What challenges have you faced with caring for patients during the COVID-19 pandemic? 3. What strategies did you use for the aforementioned challenges? 4. How did you feel when transferring the COVID-19 patients? 5. How did caring for patients with COVID-19 affect your personal life? 6. How did you cope with the changes induced by caring for COVID-19 in your personal life?
Exploratory questions	“Could you explain this more?” “Can you make this clearer?” “Can you clarify what you mean with an example?” “What do you mean?”

Each interview was conducted individually. The researchers continued conducting interviews until the point of data saturation was reached. Data saturation refers to the stage at which no new information can be obtained and coding becomes unfeasible (Lewis, 2018). After 15 interviews, data saturation was achieved; however, the researchers conducted two additional interviews with pre-hospital medical staff to ensure data replication. Notably, the heterogeneity in participants’ education levels, work experiences, and time spent working during the COVID-19 pandemic was reflected in the data saturation, indicating that each group of participants had no new data to contribute after reaching saturation.

M.J. O. and H.S., the interviewers, possess over 10 years of research experience and have completed relevant qualitative research courses, including training in interviewing, coding, and reporting. They also actively instruct students in qualitative research methods, showcasing their expertise and commitment to advancing the field. With their extensive qualifications, training, and experience, M.J. O. and H.S. are well-equipped to conduct interviews and provide valuable insights into the subject matter.

### 2.4 Data analysis

The data analysis followed a rigorous and systematic approach, utilizing the following 7 stepts of Colaizzi’s seven-step method (Edward and Welch, 2011; Morrow et al., 2015).

Step 1: The researcher (M.J. and M.D.) read a description of each person participating in the study to gain a sense of the participants. This involved carefully reviewing the data collected, such as interview transcripts or field notes, to become familiar with the participants’ experiences and perspectives.

Step 2: The researcher extracted statements with significance to the research question. These statements, often in the form of direct quotations from the participants, captured key aspects of their experiences or perspectives. These statements were referred to as “meaning units” as they contained the core meaning or essence relevant to the research question.

Step 3: The researcher began to articulate what the statements meant. This involved a process of reflection and interpretation to explore the underlying meanings embedded within the extracted statements. The researcher engaged in a thorough analysis to understand the nuances, emotions, and insights conveyed by the participants.

Step 4: Themes were created from the meanings. Based on the interpretations made in Step 3, the researcher identified common patterns, recurring ideas, or significant concepts across the meaning units. These patterns were organized into themes that captured the essence of the participants’ experiences or perspectives.

Step 5: The researcher grouped similar themes together and organized them into categories. This step involved a higher-level analysis where the researcher looked for connections and relationships among the identified themes. Themes that shared similarities or related to a broader concept were grouped together, and overarching categories were formed to provide a structured framework for organizing the data.

Step 6: Finally, the researcher integrated the results into a comprehensive description of the topic. By systematically analyzing and organizing the themes and categories, the researcher constructed a coherent and comprehensive narrative that captured the essence of the participants’ experiences or perspectives. This description aimed to provide a rich understanding of the research topic based on the data collected.

Step 7: The researcher returned to each participant to verify the results. This step involved member checking, where the researcher sought feedback from the participants to validate the accuracy and trustworthiness of the findings. This iterative process allowed participants to review and confirm the interpretation of their experiences, ensuring the research findings aligned with their viewpoints. MAXQDA software version 10 was used to analyze the data (Kuckartz and Rädiker, 2019). The final analysis was reviewed and confirmed by two additional researchers (T.M. and A.E.), enhancing the credibility and reliability of the analysis process. During the analysis, a total of 475 primary codes were initially extracted. To ensure clarity and avoid redundancy, the codes were carefully reviewed, merged, and duplicates were eliminated. As a result, the remaining codes were organized into five overarching themes that captured the essence of the data.

### 2.5 Rigor

The consolidated criteria for qualitative reporting research (COREQ) was used to guide this study (Tong et al., 2007) (Supplementary file S1). Further, the authors employed several strategies to ensure rigor in their study. They utilized triangulation by combining multiple sources of data, methods, and perspectives to provide a comprehensive understanding of the research topic. Member checking was conducted, involving the verification of findings by sharing preliminary results with participants and seeking their feedback. Peer debriefing was employed, allowing for discussions with colleagues and experts to gain valuable insights and enhance the credibility of the research. The authors addressed reflexivity by being aware of their biases and reflecting on their influences throughout the research process. They incorporated thick description, providing rich and detailed descriptions of the research context, participants, and findings to enhance credibility. Saturation was assessed, ensuring that a sufficient amount of data had been collected to capture the breadth and depth of the research topic.

### 2.6 Ethical considerations

The research project was approved by Baqiyatallah University of Medical Sciences (BUMS) (ID: IR.BMSU.REC 0.1399.133). Participants were informed that the interviews would be recorded before beginning the interviews, and their permission was requested. Participants were informed that they could withdraw at any time without penalty. The participants’ names were kept private, and the codes (P1, P2, P3, etc.) were provided.

## 3 Findings

### 3.1 Participant characteristics

The study aimed to capture the experiences of 17 pre-hospital medical staff members who had at least 1 year of experience in the field during the COVID-19 pandemic. The participants had a mean age of 39.13 years (SD = 1.60) and an average work experience of 13.8 years (SD = 1.76). Among the participants, three reported a previous positive COVID-19 test, indicating that they had contracted the disease. However, several other participants mentioned experiencing mild symptoms associated with COVID-19, such as cough, fever or chills, headache, sore throat, muscle pain, or dyspnea, either presently or in the past. Due to the intermittent and relatively mild nature of their symptoms, they had not undergone COVID-19 testing. A comprehensive overview of the participants’ characteristics can be found in Table 2. To ensure data replicability, two additional pre-hospital medical staff members were interviewed, even though data saturation had been reached with 15 participants.

**Table 2:** Sociodemographic characteristics of the participants (*N* = 15).

Participants		Educational background	Age (years)	Gender	Marital status	Pandemic work duration (month)	History of COVID-19 positive test	COVID-19 symptom history	EMT work experience (years)	Interview duration (min)
P1	EMT	Bachelor’s degree in EMS	36	Male	Married	24	No	Yes	13	40
P2	EMT	Associate’s degree in EMS	40	Male	Married	20	No	Yes	22	60
P3	EMT	Master’s degree in nursing	39	Male	Married	16	No	Yes	13	45
P4	EMT	Bachelor’s degree in EMS	33	Male	Single	12	No	Yes	11	50
P5	EMT	Master’s degree in nursing	32	Male	Married	12	No	Yes	10	55
P6	EMT	Bachelor’s degree in EMS	37	Male	Married	12	No	Yes	14	47
P7	EMT	Bachelor’s degree in EMS	34	Male	Married	14	Yes	Yes	13	53
P8	EMT	Associate’s degree in EMS	42	Male	Married	15	No	Yes	18	60
P9	EMT	Bachelor’s degree in EMS	52	Male	Married	18	No	Yes	25	60
P10	EMT	Master’s degree in nursing	47	Male	Married	15	No	Yes	23	60
P11	EMT	Bachelor’s degree in EMS	40	Male	Married	13	Yes	Yes	22	53
P12	EMT	Bachelor’s degree in EMS	45	Male	Married	12	No	Yes	5	55
P13	EMT	Associate’s degree in EMS	43	Male	Married	17	Yes	Yes	8	51
P14	EMT	Associate’s degree in EMS	39	Male	Married	12	No	Yes	6	42
P15	EMT	Associate’s degree in EMS	28	Male	Single	19	No	Yes	4	40
P16	EMT	Associate’s degree in EMS	38	Male	Married	18	No	Yes	8	39
P17	EMT	Bachelor’s degree in EMS	52	Male	Married	12	No	Yes	28	56

The study identified five main themes, namely workload and resilience, damage, lack of control, under preparedness, and post-traumatic growth (Table 3).

**Table 3:** Themes, subthemes, and codes.

Themes	Sub-themes	Codes
Workload and Resilience	Excessive workload	Increased ambulance dispatch, overwhelming emergency calls, educational needs, never-ending telephone counseling, staff shortage
	Sacrifice	Taking gloves off, removing gowns, wearing a simple surgical mask rather than an N95 respirator, removing goggles
	Fragile resilience	Infection with COVID-19, reduction in workforce, the dilemma between whether to continue or quit the work.
Damages	Mental distress	Fear of becoming infected, concern about the virus spreading to family members, relatives, or strangers, and worry about near death
	Social stress	Being far from family members, relatives, and friends, being isolated at work, being isolated at home
	Physical harm	Difficulty in breathing, poor lung CT scan, death of staff or family members
A lack of control’	Ambiguity	Being ambiguous about the disease, unclear about prevention methods, vague about therapy, and vague about complications
	Difficult pre-hospital management	Failure of all existing medication therapy, the ineffectiveness of alternative treatments, and the ineffectiveness of vaccinations
	Becoming more lethal	New variations with a higher mortality rate and new characteristics with more serious complications
Under-preparedness	Delayed action	Delayed public announcement, delayed medical staff announcement, delayed prompt action
	Early PPE Shortage	having insufficient PPE and expensive prevention tools like hand wash agents
Post-traumatic growth	Professional image	Patients’ prayers for EMTs, gratitude to EMTs in media or by high-ranking community authorities, people’s support
	Professional advancement	Raised salary, some solved professional issues, sense of empowerment in coping strategy, surge management, and overcoming crisis

### 3.2 Theme 1: workload and resilience

The COVID-19 pandemic brought about unexpected surges in ambulance demand, placing a tremendous burden on pre-hospital medical staff. They found themselves continuously transferring critically ill patients to healthcare facilities, working with limited resources, and facing high demands, which strained their resilience.

#### 3.2.1 Sub-theme I: excessive workload

The prevalence of infections resulted in a significant rise in the number of ambulance dispatches. Each day, a large number of individuals with fever and respiratory symptoms required ambulance services. Ambulances were constantly engaged in transporting critical patients. Additionally, pre-hospital medical staff had to work overtime to compensate for staffing shortages. In addition to their pre-hospital care responsibilities, they also provided telephone counseling to non-critically infected patients who were quarantined at home. Two participants expressed their experiences:

*“We were overwhelmed by the daily influx of patients requiring transportation. Keeping up with the demand was a challenge.” (P2).*

*“We had to work long hours and provide counseling over the phone. It was physically and mentally exhausting.” (P13).*

Staff shortages occurred due to infection-related contamination. Some staff members were either hospitalized or placed in home quarantine, leaving the remaining workforce to face an overwhelming workload. As a result, pre-hospital medical staff had to extend their working hours to compensate for the staffing gap. One participant shared:

*“As the Coronavirus gradually reduced our workforce by infecting our colleagues, pre-hospital medical staff had no choice but to work extra hours to cover the staffing shortfall.” (P13).*

#### 3.2.2 Sub-theme 2: sacrifice

Due to prevailing negative attitudes toward COVID-19 patients and their relatives within certain communities, the infection became a taboo topic. People would flee or avoid areas where COVID-19 patients were present or had close relatives. It was disconcerting for pre-hospital medical staff to be seen wearing PPE such as gloves, face shields, gowns, goggles, and N95 masks in these neighborhoods, as it signaled the presence of a COVID-19-infected individual. Some patients and their family members hesitated to call emergency services (e.g., dialing 115) due to the associated taboos. Consequently, pre-hospital medical staff often had to remove certain parts of their PPE to navigate these taboos. Two participants expressed:

*“If a family was infected with COVID-19, other neighbors would want to leave the apartment or block to ensure their safety or they would view the infected family negatively.” (P1).*

*“To avoid the stigma, we often had to remove most of our PPE while in the patients’ neighborhood and transport them wearing masks and gloves only!” (P8).*

#### 3.2.3 Sub-theme 3: fragile resilience

Pre-hospital medical staff faced numerous challenges in their work, including staff shortages, consecutive long shifts, inadequate safety measures, stressful situations, and high mortality rates. While they managed to overcome these obstacles, the hardships and difficulties they encountered had the potential to weaken their resilience and determination at any given time, causing them to consider giving up the fight against the pandemic. In some cases, family members of individuals with underlying chronic illnesses would pressure them to leave the profession in order to prioritize their own safety. However, despite the difficulties, these dedicated professionals persisted in providing pre-hospital services. Two participants expressed their perspectives:

*“We were torn between prioritizing our own safety and staying on duty. However, our unwavering commitment compelled us to continue offering our services in the face of any adversity.” (P9).*

*“The stressful circumstances, inadequate equipment and staff, and lack of support were enough to make us contemplate leaving the job.” (P5).*

### 3.3 Theme 2: damage

Pre-hospital medical staff experienced significant physical, psychological, and social impacts as a result of COVID-19. Among them, three individuals tested positive for COVID-19 and suffered from severe psychological issues and physical injuries, while others exhibited symptoms such as dyspnea, fatigue, and myalgia. Sadly, some pre-hospital medical staff succumbed to the disease after experiencing severe conditions. The second theme encompassed psychiatric distress, social stress, and physical harm.

#### 3.3.1 Sub-theme 1: psychiatric distress

The pre-hospital medical staff not only grappled with concerns about their own vulnerability to infection but also harbored anxiety about potentially transmitting the disease to their loved ones. The high mortality rates further exacerbated their distress.

*“Several of our colleagues had family members who were hospitalized and, tragically, some even passed away! I was primarily worried about the possibility of spreading the infection to my wife and children.” (P7).*

*“We witnessed a significant number of patients we transported by ambulance succumbing to the disease in hospitals later on! Such news deeply impacted our emotional well-being!” (P8).*

#### 3.3.2 Sub-theme 2: social stress

In order to prevent the transmission of infection to their immediate family members, pre-hospital medical staff resorted to self-isolating either within a designated room in their homes or in public spaces such as their workplaces. Additionally, due to their extensive interaction with individuals afflicted by COVID-19, these personnel were often stigmatized as potential carriers of the virus, resulting in people actively avoiding their presence. Consequently, pre-hospital medical staff found themselves distanced from both their loved ones and the general public, a measure aimed at maintaining social distance but one that also introduced significant social stress into their lives.

Participant 11 expressed concerns about the proximity to family members within their household, stating, *“The thought of being in close proximity to my family members in the house worries me. As a precautionary measure, I isolate myself in a separate room at all times. Additionally, after completing my shifts, I ensure to change my protective suit in the parking lot.”*

Participant 2 highlighted the experience of being avoided by people in their neighborhood due to the perception that they were potential carriers of the disease. He shared, *“I observed that individuals within our neighborhood who were familiar with my profession actively avoided me, as they held the belief that I posed a risk of transmitting the disease.”*

#### 3.3.3 Sub-theme 3: physical harm

##### 3.3.3.1 Physical impact

Pre-hospital medical staff faced significant physical challenges arising from their round-the-clock exposure to infected patients, extended working hours, sleep deprivation, and inadequate nutrition. Tragically, some of these dedicated professionals succumbed to organ or multi-organ failure after contracting COVID-19. Common physical symptoms experienced by pre-hospital medical staff included fever, shortness of breath, excessive sweating, bruising, body pain, and persistent coughing. Moreover, the toll of the virus extended beyond the staff themselves, with numerous family members also falling ill and, in certain cases, even losing their lives.

Participant 13 vividly captured the sense of vulnerability within the profession, sharing, *“At any given moment, all of us were susceptible to disease and death. Several of our colleagues or their family members have already been infected or, tragically, passed away.”*

Participant 11 conveyed the lasting impact on their respiratory system, revealing, *“Even five months after contracting the disease, I continue to experience coughing. My respiratory system has suffered significant damage.”*

### 3.4 Theme 3: a lack of control

Despite the widespread vaccination efforts (Di Mauro et al., 2022), the unpredictable nature of COVID-19 persisted due to the emergence of new virus mutations and the development of sub-variants. This theme encompasses the notions of “ambiguity,” “ineffective treatment,” and “increased lethality.”

#### 3.4.1 Sub-theme 1: ambiguity

The lack of comprehensive understanding regarding COVID-19 has contributed to its global spread. The novel virus remains enigmatic, with limited specific information available. Participant 7 emphasized the vast unknowns surrounding the virus, stating, *“Every aspect of the virus, from its origins to its control, remains a mystery. The virus exhibits periodicity, and its mutations further complicate matters.”* Participant 5 echoed this sentiment, expressing uncertainty about the future of the ongoing situation.

#### 3.4.2 Sub-theme 2: difficult pre-hospital management

Managing critical cases in the pre-hospital setting proved arduous. Despite employing various medical treatment techniques, the patients’ conditions remained unmanageable, necessitating their intubation and mechanical ventilation. Participant 5 highlighted the ineffectiveness of medications administered to severely ill individuals, sharing, “*None of the drugs administered to these critically ill patients proved effective. I also worked in a hospital concurrently and realized that only divine intervention could treat such severe cases.”* Participant 7 further reflected on the gravity of the situation, expressing concern about the potential shortage of oxygen and ventilators for critically ill patients. *“The situation was so bad that we were afraid that we would not further able to provide oxygen and ventilators for the critical patients.”*

#### 3.4.3 Sub-theme 3: becoming more lethal

##### 3.4.3.1 Escalating lethality

In recent waves, notably the Delta variant, COVID-19 demonstrated an increased lethality compared to previous strains. Surprisingly, a significant number of young individuals fell critically ill despite their anticipated resilience. Participant 15 expressed the helplessness experienced in the face of the virus, stating, *“There seems to be no solution to this virus. I ponder when this disease will finally abate.”* Participant 12 shared the alarming nature of their calls during the Delta wave, with critically ill patients teetering on the brink of death, saying, *“All of our calls in the Delta wave were from critically ill patients, and in some dispatches, critically ill patients were on the verge of death.” (P 12).*

### 3.5 Theme 4: under preparedness

The theme of under preparedness highlights the community’s initial lack of readiness to effectively mitigate the disease’s spread when the outbreak began. Consequently, the infection rapidly disseminated throughout the country. This theme encompasses the sub-themes of “delayed action” and “PPE shortage.”

#### 3.5.1 Sub-theme 1: delayed action

In response to the emergence of the pandemic in China, flights between Iran and countries experiencing conflicts continued, providing an entry point for the virus. There was a delay in informing the public about the new infection, contributing to the subsequent outbreak within the country. The lack of anticipation regarding a pandemic may have contributed to this delay. Additionally, the experiences of pre-hospital medical staff revealed that, despite periodic quarantine requirements during various waves, compliance with quarantine rules was hindered by the country’s challenging economic circumstances, exacerbated by severe sanctions.

Participant 2 recalled the initial perception of the situation, stating, *“Initially, the condition was not regarded as serious. Officials initially claimed that the infection was unlikely to spread from China to other countries.”* Participant 4 highlighted the economic realities faced by individuals, explaining that the need to work, even during quarantine periods, stemmed from the financial obligations associated with their shops and the need to pay monthly rent. *“If people close their shops, how can they pay the monthly rent of the shop? They have to work under any conditions, even during quarantine time.” (P4).*

#### 3.5.2 Sub-theme 2: early PPE shortage

In the early stages of the pandemic, health facilities faced a shortage of PPE due to insufficient planning. Additionally, the high prices of PPE and the country’s economic challenges further exacerbated the situation, making it difficult for people to purchase adequate protective gear.

Participant 3 shared the scarcity of PPE during the initial waves, stating, *“There was an insufficient supply of PPE in the early stages. We had to personally purchase them.”* Participant 11 elaborated on the restrictions imposed on PPE usage, explaining that staff members were provided with limited quantities of PPE and were not permitted to exceed their allotted quota. *“PPE was supplied to the staff according to quotas, and we were not permitted to utilize more than our limit.” (P11).*

### 3.6 Theme 5: post-traumatic growth

Despite the challenging circumstances faced during the pandemic, first responders experienced positive outcomes and personal growth. They received recognition, dignity, and respect from society, similar to other healthcare workers. This theme encompasses the sub-themes of “professional image” and “professional advancement.”

#### 3.6.1 Sub-theme 1: professional image

The commitment and contributions of pre-hospital medical staff in saving lives were witnessed by the public. Their sacrifices were readily apparent, and during the outbreak, people endeavored to support healthcare workers in any way possible. This support manifested through emotional displays, provision of PPE, and distribution of food packages. Numerous individuals, as well as authorities and the media, expressed gratitude and admiration for the sacrifices made by healthcare providers, including pre-hospital medical staff.

Participant 15 reflected on the unprecedented emotional treatment received, stating, *“We observed that people generally appreciated and expressed more emotions towards us, which was remarkable.”* Participant 9 highlighted the praise and gratitude conveyed by officials and the media toward healthcare workers. He said: *“During the pandemic, officials in the media praised and thanked the health care workers.” (P9).*

#### 3.6.2 Sub-theme 2: professional advancement

Pre-hospital medical staff, alongside other healthcare professionals, played a crucial role in responding to the COVID-19 pandemic, extending their capabilities beyond normal limits. The experience of managing the pandemic empowered pre-hospital medical staff in terms of coping strategies, managing increased demands, and patient care.

Participant 2 expressed satisfaction and delight in witnessing positive reactions from authorities, stating, *“Although we are currently engaged in a genuine war, the positive responses from the authorities uplift us.”* Participant 3 recognized the pandemic’s challenges but also acknowledged the valuable lessons learned, stating, *“Despite the numerous challenges, the pandemic has been a source of growth. It has enhanced our ability to provide pre-hospital services, and I feel a boost in my self-esteem.”*

## 4 Discussion

In this study, we explored the experiences of Iranian pre-hospital medical staff during the COVID-19 pandemic, identifying five key themes: workload and resilience, damage, lack of control, under preparedness, and post-traumatic growth. The inequality between call volumes and responses affected staff resilience, leading to a heavy burden and damage. The initial lack of control and unpreparedness worsened the situation. However, first responders’ sacrifices in managing the increased demand for emergency services garnered community respect and contributed to professional growth.

The first theme highlights that when the workload significantly exceeded capacity and staff numbers declined due to COVID-19 infections, the resilience of the staff was compromised. These findings align with a previous studies indicating a surge in demand for ambulance services during the COVID-19 pandemic (Kinross et al., 2020), with increased calls related to COVID-19 cases and a decrease in other emergencies like road accidents (Lerner et al., 2020), which have had a profound impact on their resilience (Lai et al., 2020; Sun et al., 2020). The pre-hospital system in Iran has been heavily burdened by the outbreak, with pre-hospital medical staff playing a crucial role in patient management, including screening, primary care, and hospital transfers (Hadian et al., 2022).

To mitigate the negative impact of increased workload and fragile resilience, studies suggest strategies such as resilience, determination, humor, competence, and emotional tolerance (Piotrowski et al., 2021). In Iran, the government requested overtime and extended shifts from existing staff to address increased workload. They also recruited recent nursing graduates and medical personnel to fill workforce gaps in pre-hospital emergency care (Hadian et al., 2022). The authors suggest teaching adaptive strategies to enhance the resilience of pre-hospital staff. Moreover, implementing effective triage systems can address call volume inequalities, while workload analysis optimizes resource allocation (Marks, 2022).

A portion of our findings emphasized the adverse effects of COVID-19-related stigma on the staff, corroborated by other studies (Turner-Musa et al., 2020; Bhanot et al., 2021). To address COVID-19-related stigma, our research suggests strategies such as public awareness campaigns, media engagement, community dialogs, empathy education, and legal protections against discrimination. These measures could promote public education, compassionate treatment, and a supportive society (Valeri et al., 2021).

The “damage” theme explores challenges faced by pre-hospital medical staff, encompassing physical injury, psychological discomfort, and social stress. Positive-test participants experienced these challenges more frequently. A previous study (Higginson et al., 2020) highlighted the physical health risks faced by pre-hospital medical staff during the early days of the COVID-19 pandemic, attributed to poor recognition of the disease and inadequate PPE. From author’s opinion, protecting their safety requires prioritizing the availability and appropriate use of adequate PPE, providing regular infection control training, and implementing comprehensive occupational health and safety protocols (Marques et al., 2020).

Our study revealed that pre-hospital staff suffered from stress, in line with our findings, Piotrowski et al. (2021) highlighted the heightened stress, exhaustion, and worry experienced by pre-hospital medical staff (Piotrowski et al., 2021), emphasizing the need for tailored mental health support programs. According to researchers, regular check-ins, access to counseling services, and resilience-building techniques can be crucial steps for mitigating psychological challenges.

In addition to mental stress and physical harm, pre-hospital staff also experienced social stress as another significant factor of concern, which confirmed by other studies (Alahdal et al., 2020; Vindrola-Padros et al., 2020; Alqahtani et al., 2021). According to researchers, prioritizing initiatives like virtual communication platforms is crucial to address the social and emotional needs of pre-hospital staff.

Our third theme, “a lack of control,” highlights the challenges in managing the COVID-19 pandemic due to the unknown nature of the virus and absence of effective treatments. The global healthcare systems, including Iran’s, have been heavily impacted by the COVID-19 pandemic. Effectively managing this crisis necessitates the adoption of novel strategies worldwide (Zargham et al., 2021). Chen et al. (2021) also emphasized these challenges and the impact of virus mutations (Chen et al., 2021). The emergence of new variants, like the Delta strain, poses significant threats with higher infectivity and viral load (Yu et al., 2021). Based on researcher perspectives, it is crucial for healthcare professionals to stay updated on research and guidelines to adapt infection control and treatment strategies accordingly, as per researcher perspectives (Majid et al., 2011).

The feeling of helplessness and loss experienced by caregivers due to a lack of control over the pandemic and infection control is another significant aspect to consider (Iheduru-Anderson, 2021). Comprehensive training, support, and regular updates on guidelines are crucial in addressing the challenges faced by healthcare professionals in managing the pandemic and ensuring they have the necessary knowledge, skills, and resources (Frenk et al., 2022).

As our findings reflected, the lack of preparedness in managing the ongoing crisis has multifactorial causes. One contributing factor is the lack of collaboration between authorities, scientific institutions, and the media (Ruiu, 2020). Encouraging transparency, trust, and collaboration can enhances preparedness and response capabilities while addressing barriers such as conflicting news, delays in official announcements, paternalistic attitudes, and political ambiguity that hinder population awareness and preparedness (Ruiu, 2020). Therefore, healthcare professionals and authorities should prioritize clear and consistent communication to the public, providing up-to-date information, guidance, and instructions. Open and transparent communication channels can help build trust, address misconceptions, and foster public compliance with preventive measures (Lee and Kwak, 2012).

Our findings highlighted the positive impact of the COVID-19 pandemic on pre-hospital medical staff, including increased visibility and recognition within society. Notably, they have received positive feedback, social respect, and increased pay. Other researcher reported that the acknowledgment by the general population, the media, and government authorities further validates the professionalism and dedication of these front-line responders (Liu et al., 2020; Sun et al., 2020).

### 4.1 Limitation

Our study focused on pre-hospital services provided by pre-hospital medical staff in Tehran, the capital of Iran. It is possible that the conditions of pre-hospital care in other cities with limited facilities may be more challenging. One limitation of our study is the potential influence of the researcher’s preconceived notions on the results. To mitigate this effect, the researcher took precautions to set aside their prior knowledge and biases at the beginning of the study, thus minimizing their influence on the findings. Furthermore, it is worth considering the economic, cultural, and social differences between Iran and other countries.

## 5 Conclusion

The surge in pre-hospital treatment requests during the COVID-19 pandemic has significantly impacted the workload and well-being of medical staff. This imbalance has led to mental, social, and physical challenges. Despite the hardships, their sacrifices have improved their professional image and garnered increased respect. To support pre-hospital staff, resources and support should be provided, including adequate staffing, sufficient PPE, and well-being programs. Fostering a culture of accountability and providing necessary training further enhances their performance. Individuals previously infected with COVID-19 experienced distress and transmission concerns, warranting further studies for comparison.

## Data availability statement

The original contributions presented in the study are included in the article/Supplementary material, further inquiries can be directed to the corresponding authors.

## Author contributions

MJ-O, STM, and AE conceived the study design. HS, MJ-O collected the data, conducted the data analysis, and wrote the manuscript. STM conducted the data analysis. MJ-O, STM, AE, MD, and HS have made a considerable contribution to the study design, data analysis, interpretation of results, and writing of the manuscript. All authors contributed to the article and approved the submitted version.

## Ethics statement

The studies involving humans were approved by the Baqiyatallah University of Medical Sciences (BUMS) (ID: IR.BMSU.REC.1399.133). The studies were conducted in accordance with the local legislation and institutional requirements. The participants provided their written informed consent to participate in this study.
